# Role of the Lactide:Glycolide Ratio in PLGA Nanoparticle Stability and Release under Lysosomal Conditions for Enzyme Replacement Therapy of Lysosomal Storage Disorders

**DOI:** 10.3390/jfb14090440

**Published:** 2023-08-25

**Authors:** Maria del Moral, Maximilian Loeck, Eameema Muntimadugu, Guillem Vives, Vy Pham, Peter Pfeifer, Giuseppe Battaglia, Silvia Muro

**Affiliations:** 1 Institute for Bioengineering of Catalonia (IBEC), Barcelona Institute for Science and Technology, 08028 Barcelona, Spain; 2 Applied Materials Chemistry Master Program (M.d.M) and Biomedicine Doctorate Program, University of Barcelona, 08007 Barcelona, Spain; 3 Institute for Bioscience and Biotechnology Research (IBBR), University of Maryland, College Park, MD 20742, USA; 4 Nanoscience and Nanotechnology Degree Program, Autonomous University of Barcelona, 08193 Bellaterra, Spain; 5 Department of Chemical and Biomolecular Engineering, University of Maryland, College Park, MD 20742, USA; 6 Institution of Catalonia for Research and Advanced Studies (ICREA), 08010 Barcelona, Spain

**Keywords:** lysosomal storage disorder, enzyme replacement therapy, hyaluronidase, poly(lactide-co-glycolide) nanoparticles, copolymer ratio, nanoparticle stability, enzyme release

## Abstract

Prior studies demonstrated that encapsulation in poly(lactic-co-glycolic acid) (PLGA) nanoparticles (NPs) enhanced the delivery of enzymes used for replacement therapy (ERT) of lysosomal storage disorders (LSDs). This study examined how the copolymer lactide:glycolide ratio impacts encapsulation, physicochemical characteristics, stability, and release under lysosomal conditions. Hyaluronidase, deficient in mucopolysaccharidosis IX, was encapsulated in NPs synthesized using 50:50, 60:40, or 75:25 lactide:glycolide copolymers. All NPs had diameters compatible with cellular transport (≤168 nm) and polydispersity indexes (≤0.16) and ζ-potentials (≤−35 mV) compatible with colloidal stability. Yet, their encapsulation efficiency varied, with 75:25 NPs and 60:40 NPs having the lowest and highest EE, respectively (15% vs. 28%). Under lysosomal conditions, the 50:50 copolymer degraded fastest (41% in 1 week), as expected, and the presence of a targeting antibody coat did not alter this result. Additionally, 60:40 NPs destabilized fastest (<1 week) because of their smaller diameter, and 75:25 NPs did not destabilize in 4 weeks. All formulations presented burst release under lysosomal conditions (56–78% of the original load within 30 min), with 50:50 and 60:40 NPs releasing an additional small fraction after week 1. This provided 4 weeks of sustained catalytic activity, sufficient to fully degrade a substrate. Altogether, the 60:40 NP formulation is preferred given its higher EE, and 50:50 NPs represent a valid alternative, while the highest stability of 75:25 NPs may impair lysosomes. These results can guide future studies aiming to translate PLGA NP-based ERT for this and other LSDs.

## 1. Introduction

Lysosomes play a central role in maintaining cellular homeostasis by intersecting with multiple biological functions, including cellular metabolism, recycling, signaling, defense, and cell death [1,2,3,4]. As such, alterations in the normal lysosomal function can lead to highly debilitating and often lethal pathological conditions [4,5,6]. An example is lysosomal storage disorders (LSDs), a group of ≈60 congenital diseases caused by a genetic deficiency of lysosomal components, most commonly enzymes [6,7,8]. As a result, LSDs are characterized by the accumulation of undigested substrates within cellular lysosomes, which affects multiple cell types and tissues, causing multi-organ dysfunctions [7,8]. The onset of LSD symptoms ranges widely: less severe forms of disease that affect peripheral organs appear in infancy or adulthood, while more severe forms of disease that additionally affect the central nervous system (CNS) appear early after birth [9,10]. In all cases, symptoms markedly weaken patients and contribute to premature morbidity and mortality [9,10].

The first-line treatment for LSDs is enzyme replacement therapy (ERT) using intravenous (i.v.) infusion of recombinant enzymes to restore deficient function [11,12]. This strategy has resulted in ≈20 clinical products currently used to treat non-CNS forms of Gaucher disease, Fabry disease, Pompe disease, acid sphingomyelinase deficiency, and several mucopolysaccharidoses (MPSs) [11,12]. Nevertheless, ERT is limited by side effects and the development of “resistance”, which typically arises from immune detection and/or systemic activity of recombinant enzymes before reaching lysosomes, since these products are injected as “naked” proteins [11,13,14]. Furthermore, recombinant enzymes cannot cross the blood–brain barrier (BBB), a highly selective membrane separating the general circulation from the CNS, which limits treatment for severe LSD forms affecting the CNS [11,13,14].

Various approaches are being explored to help with these aspects. One strategy aims to provide recombinant enzymes with targeting elements to enhance their transport to disease sites, including the CNS, while another strategy aims to protect, hide, and/or control the release of enzymes by encapsulation [15,16]. The first approach includes mostly antibody-derived or ligand-derived peptides tagged onto enzymes using chemical conjugation or recombinant fusion [15]. In the second approach, enzymes are carried by therapeutic nanocarriers whose physicochemical properties can be tuned to optimize pharmacokinetics and pharmacodynamics [16]. These nanosystems can also display targeting elements [11,16]. Nanocarriers under investigation for lysosomal ERT include exosomes and extracellular vesicles [17], liposomes and other lipid-based systems [18], and polymer-based nanoparticles (NPs) [19]. Among the latter class, NPs made from poly(lactic-co-glycolic acid) or PLGA have drawn attention for lysosomal ERT because of various advantages. For instance, PLGA is commercially available, can be synthesized with high purity and reproducibility, and is approved for clinical use [20,21]. PLGA NPs can be fabricated using various colloidal techniques such as emulsification–diffusion, emulsification–evaporation, nanoprecipitation, etc. [22,23,24,25]. PLGA can be degraded in the body by hydrolytic cleavage of ester bonds, rendering lactic acid and glycolic acid, common biological byproducts, easily metabolized by cells [21,26,27]. Also, PLGA degradation in lysosomes can restore the acidic pH of this organelle in cells affected by LSDs, whose pH is otherwise altered (less acidic), representing a beneficial effect of this material [28,29,30]. 

For these reasons, several studies have explored PLGA NPs for lysosomal ERT, including formulations loaded with acid sphingomyelinase for treatment of Niemann–Pick disease types A or B, hyaluronidase (HAse) for MPS type IX, iduronate-2-sulfatase for MPS type II, palmitoyl-protein thioesterase-1 for neuronal ceroid lipofuscinosis 1, galactosylceramidase for Krabbe disease, and acid α-glucosidase for Pompe disease [31,32,33,34,35,36,37,38,39,40,41]. Some of these PLGA NP formulations additionally displayed targeting elements for receptor-mediated transport across the BBB and/or inside target cells, such as intercellular adhesion molecule 1 (ICAM-1) antibodies, angiopep-2 oligopeptide, transferrin-binding peptide Tf2, or g7 glycopeptide [31,32,33,34,36,37,38,39,40]. All these studies have demonstrated enhanced delivery and/or effects of lysosomal enzymes in cell culture and/or animal models [31,32,33,34,35,36,37,38,39,40,41]. However, their delivery efficacy and associated effects varied among different formulations [31,32,33,34,35,36,37,38,39,40,41]. This was expected since each formulation used a different enzyme cargo, targeting receptor, disease model, PLGA copolymer, and/or NP-synthesis method [31,32,33,34,35,36,37,38,39,40,41], for which more comparative investigations are needed.

Among these factors, PLGA’s lactide:glycolide ratio is of key relevance since it is known to impact the overall copolymer hydrophobicity and, thus, physicochemical features such as NP encapsulation efficiency, size, stability, degradation, and cargo release [42,43,44,45,46,47]. These parameters are important since they are expected to influence: (a) the ability of PLGA NPs to load a sufficient amount of a hydrophilic therapeutic cargo, such as the case for lysosomal enzymes; (b) the size of NPs, so that they avoid free diffusion across membranes but are compatible with endothelial transcytosis and cellular uptake with transport to lysosomes; (c) NP stability, so that they withhold their function until reaching lysosomes; and (d) degradation to enable enzyme release within this cellular compartment [42,43,44,45,46,47]. 

Therefore, the present study focused on exploring the role of PLGA copolymer, particularly, the lactide:glycolide ratio, on the encapsulation efficiency and physicochemical properties, as well as the stability and release under lysosomal conditions, of NPs encapsulating an enzyme for lysosomal ERT. We used PLGA copolymers with either 50:50, 60:40, or 75:25 lactide:glycolide ratios to formulate NPs encapsulating HAse, the enzyme deficient in MPS type IX (OMIM 601492), as a model [48,49,50]. The results obtained provide a better understanding of the potential of PLGA NPs for lysosomal ERT and should help optimize these formulations for future applications.

## 2. Materials and Methods

### 2.1. Reagents

PLGA 50:50 (ester terminated, 55–80 kDa average molecular weight (MW)) was purchased from Evonik (Birmingham, AL, USA). PLGA 60:40 (ester terminated, 45–55 kDa average MW) and PLGA 75:25 (ester terminated, 45–55 kDa average MW) were purchased from PolySciTech (West Lafayette, IN, USA). Mouse anti-human ICAM-1 (clone R6.5) was affinity purified from a hybridoma obtained from American Type Culture Collection (Manassas, VA, USA). Bovine serum albumin (BSA) and phosphate buffer saline (PBS) were from Thermo Fisher Scientific (Waltham, MA, USA). Pierce Iodogen and ^125^Iodine (^125^I) were from Thermo Fisher Scientific (Waltham, MA, USA). BioSpin Tris Columns were from BioRad (Hercules, CA, USA). HAse Type I-S from bovine testes, 1000–2200 kDa hyaluronic acid (HA), Pluronic F68, ethyl acetate, trichloroacetic acid (TCA), and all other reagents were from Sigma-Aldrich (St. Louis, MO, USA). Gel permeation chromatography (GPC)-grade tetrahydrofuran (THF) was from Scharlab S.L. (Barcelona, Spain).

### 2.2. Labeling with ^125^Iodine

Radiotracing was used to determine enzyme encapsulation and release described below, for which HAse was labeled with ^125^I, as shown in [32]. Alternatively, where indicated, radiotracing was used to quantify the number of targeting antibodies coated on NPs [32]. For this purpose, 1 mg/mL of either HAse cargo or targeting anti-ICAM-1 was incubated with 20 µCi ^125^I for 10 min at room temperature (RT) using Pierce Iodogen. Then, unconjugated ^125^I was removed using BioSpin Tris Columns with centrifugation at 1000× *g* for 4 min. Next, incubation in 15% *v*/*v* TCA containing 2.5% BSA was used to precipitate protein, which was separated with centrifugation at 2300× *g* for 5 min. Following this, the ^125^I-HAse or ^125^I-anti-ICAM-1 contents were measured as counts per minute (CPMs) using a gamma counter (Hidex; Turku, Finland), and the respective protein concentrations were determined using a Bradford assay [32]. Data were used to determine ^125^I-HAse or ^125^I-anti-ICAM-1 specific activities (CPMs/mass), from which the number of ^125^I-HAse or ^125^I-anti-ICAM-1 molecules can be obtained using 55 kDa or 150 kDa as the known MWs for HAse or anti-ICAM-1, respectively. In all cases, ^125^I and ^125^I-labelled materials were handled in an approved facility, under proper supervision and monitoring, and in compliance with all institutional regulations.

### 2.3. NP Preparation 

PLGA NPs encapsulating HAse were manufactured using either 50:50, 60:40, or 75:25 glycolide:lactide copolymers, as described above (for simplicity, the resulting NPs were, respectively, called 50:50 NPs, 60:40 NPs, or 75:25 NPs). A previously described double emulsion and solvent evaporation technique was used [32]. Briefly, to form a primary emulsion, an aqueous solution containing 4 mg/mL protein was added dropwise and under stirring to an organic solution containing 18 mg/mL copolymer in ethyl acetate. The protein solution contained a 2 HAse to 1 BSA mass ratio, as this BSA proportion was recently reported to optimize HAse encapsulation [32]. This emulsion was homogenized with stirring at 1400 rpm for 30 s, followed by sonication on ice using a Fisherbrand™ Model 120 Sonic Dismembrator (Fisher Scientific, Hampton, NH, USA). To form a secondary emulsion, this emulsion was then added dropwise and under stirring to a solution containing 2% Pluronic F68 in water. This was homogenized with stirring at 1250 rpm for 1 min, followed by sonication on ice and solvent removal using overnight evaporation under stirring [32]. NPs were collected with centrifugation at 14,100× *g* for 15 min at RT using an Avanti J-26 XP centrifuge with a J-12 rotor (Beckman Coulter, Pasadena, CA, USA). 

### 2.4. NP Coating with Targeting Antibody

The NPs described above were either used without a targeting coat or further coated with an antibody that targets ICAM-1. For this purpose, NPs were mixed at RT for 2.5 h with 1 mg/mL anti-ICAM-1, as in our prior study [32]. This concentration favors antibody outward display on NPs due to a preferential interaction with polymeric surfaces through its Fc region [51]. The non-coated antibody was then separated from coated NPs using centrifugation at 12,000 rpm (Eppendorf Centrifuge 5424 R), followed by NP resuspension in PBS containing 1% BSA and sonication [32]. 

### 2.5. NP Characterization

All NPs were characterized by dynamic light scattering (DLS) using the Zetasizer Ultra instrument and analyzed with the respective ZS XPLORER software (Malvern Panalytical, Worcestershire, UK). NP hydrodynamic size and polydispersity index (PDI) were measured by diluting samples in PBS, while ζ-potential was measured by diluting samples in deionized water. 

For visualization using scanning electron microscopy (SEM), NPs were spread over slabs, dried under vacuum, coated with a gold layer in a cathodic evaporator, and finally observed using an SEM instrument from JEOL (Tokyo, Japan).

For NPs coated with ^125^I-anti-ICAM-1, the ^125^I content was measured using a gamma counter to render the number of antibody molecules in the NP preparation, as described above in Section 2.2. This was performed after incubating coated NPs in 50% serum to mimic physiological conditions and better reflect the coat expected for NPs in biological environments where targeting is intended. DLS was used to measure the number of NPs in the preparation so that the average number of antibody molecules per NP could be obtained, as in our prior study [32].

### 2.6. Encapsulation Efficiency

The encapsulation efficiency (EE) of PLGA NPs was determined using the direct method, as described [32]. For this purpose, NPs encapsulating ^125^I-HAse were centrifuged at 10,000 rpm for 10 min to separate the enzyme in the pellet. The ^125^I-HAse content of this pellet was then measured using a gamma counter after the removal of the supernatant and EE was calculated as a percentage, as follows:

$$\%EE=\frac{HAse\;in\;NC\;pellet\;(µg)}{HAse\;addrd\;during\;NC\;preparation\;(µg)}\times 100$$

where HAse µg was calculated using ^125^I-HAse specific activity (CPMs/mass), described in Section 2.2.

### 2.7. NP Stability and Enzyme Release under Lysosomal Conditions

PLGA NPs encapsulating ^125^I-HAse, either with or without anti-ICAM-1 coating, were incubated for 30 min to 4 weeks at 37 °C under gentle shaking in artificial lysosomal fluid (ALF). The ALF was pH 4.5 and contained 0.525 mM magnesium chloride, 54.93 mM sodium chloride, 0.5 mM disodium hydrogen phosphate, 0.275 mM sodium sulfate, 0.871 mM calcium chloride dihydrate, 0.262 mM sodium citrate dihydrate, 150 mM sodium hydroxide, 108.3 mM citric acid, 0.786 glycine, 0.391 mM sodium tartrate dihydrate, 0.759 mM sodium lactate, and 0.782 mM sodium pyruvate. Similar to a previous study [32], at indicated time points, NP samples were analyzed using DLS, as described in Section 2.5, to determine possible changes in NP size and PDI. Thereafter, NPs were separated using centrifugation at 12,000 rpm for 15 min, leaving released ^125^I-HAse in the supernatant. The pellet was dissolved in THF and analyzed using GPC to determine polymer MW and discern its degradation compared to control polymers. GPC measurements were run at 30 °C and 1 mL/min eluent flow through a PLgel 5 µm MIXED-D 300 × 7.5 mm column on an Agilent 1260 Infinity II instrument (Santa Clara, CA, USA). This instrument was equipped with a dual-angle light scattering detector, a refractive index detector, and a viscometer. Calibration was performed with the Agilent EasiVial polystyrene calibration standard series. Finally, ^125^I-HAse release from NPs was quantified in the supernatant using a gamma counter, before and after TCA precipitation, as described in Section 2.2. The difference between the ^125^I content measured before and after TCA precipitation corresponds to the presence of free ^125^I and short, non-precipitable ^125^I-HAse-derived peptides, which are is interpreted as ^125^I-HAse degradation. 

### 2.8. Released Enzyme Activity 

Experiments similar to those described in Section 2.7 were conducted using PLGA NPs encapsulating non-labeled HAse. NPs were lyophilized overnight at 0.140 mbar and −45 °C, resuspended in PBS, sonicated, and incubated in acetonitrile for 30 min at RT to dissolve NPs and extract HAse. After this, samples were centrifuged at 10,000 rpm for 10 min so that the polymer-containing supernatant was discarded, and the protein-containing pellet was resuspended in PBS and centrifuged again. The protein content in the supernatant and the remaining pellet (dissolved in 0.1 N NaOH) were mixed to measure enzyme activity using the Worthington™ Hyaluronidase Assay, as described in [32]. Briefly, samples were incubated for 10 min at 37 °C with 0.4 mg/mL HA (HAse’s substrate) at pH 5.3. Then, the optical density of this solution was measured at 540 nm and compared to a control HA solution and a HAse standard curve of known activity.

### 2.9. Statistics

Data were calculated as mean ± standard error of the mean (SEM) from *n* ≥ 3, and statistical significance was determined as *p* < 0.05. ANOVA followed by Tukey’s test was used for multiple comparisons, while two-group comparisons were assessed using Student’s *t*-test. All statistics were calculated using GraphPad Prism^®^ version 8 (GraphPad Software; San Diego, CA, USA).

## 3. Results and Discussion

### 3.1. Characterization of PLGA NPs Encapsulating HAse

Our recent publication compared enzyme encapsulation vs. enzyme coating using 50:50 PLGA NPs [32]. The results demonstrated that, compared with the naked enzyme, both formulations enhanced enzyme delivery in cell culture and mice [32]. The encapsulating formulation surpassed the coated one, mainly due to a higher enzyme load per NP [32]. Those NPs were lyophilized and reconstituted without major changes [32]. They were also stable as a suspension for the longest time points tested: at least 8 weeks under storage conditions (4 °C in PBS) and at least 2 days under physiological conditions (37 °C and 50% serum) [32]. In addition, 50:50 PLGA NPs encapsulating HAse were successfully coated with anti-ICAM-1 antibody for targeting, bound specifically to cells in culture, were internalized by cells and trafficked to lysosomes, and displayed catalytic activity for up to 4 weeks under lysosomal conditions (37 °C and pH 4.5 in ALF) [32]. However, the pattern of NP stability, copolymer degradation, and enzyme release were not previously assessed. Consequently, here, we aimed to investigate these parameters. In addition, because of the well-known role of the lactide:glycolide ratio in PLGA properties [42,43,44,45,46,47], we also compared 50:50 PLGA NPs to NPs obtained using 60:40 and 75:25 PLGA copolymers. 

**Figure 1:**
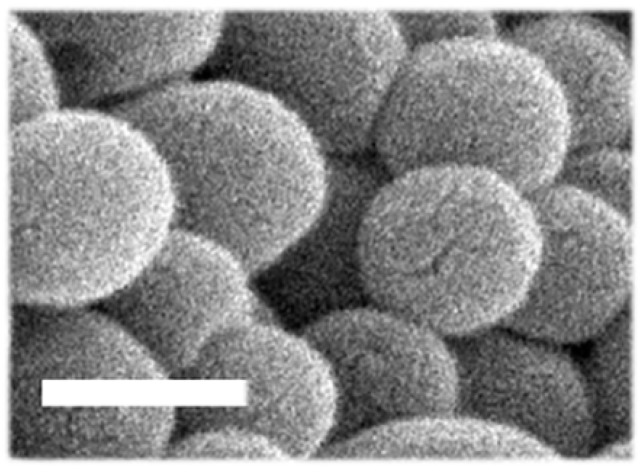
Morphology of PLGA NPs. NPs prepared using 50:50 lactide:glycolide PLGA copolymer and observed using scanning electron microscopy (SEM). Scale bar = 200 nm.

As shown in Table 1, using the same double-emulsion and solvent evaporation method, the 50:50 NP formulation reproduced well our prior data [32]. This NP formulation presented a 167.8 nm hydrodynamic diameter, as measured using DLS, which corresponded well to their spherical shape and size observed using SEM, also showing a smooth surface (Figure 1). Their PDI was 0.14 and their ζ-potential was −38.4 mV, as previously shown [32]. As for the enzyme content of these NPs, we found 20.4%EE, reproducing well our prior data [32], which is equivalent to 400.9 HAse molecules/NP or 1.6 µg HAse per mg polymer. Furthermore, 60:40 PLGA NPs had a 121.4 nm hydrodynamic diameter, smaller than the 50:50 and 75:25 formulations, where the latter had 164.7 nm and, thus, was similar to 50:50 NPs (Table 1). As for 50:50 PLGA NPs, the other two formulations were also monodispersed (0.12 and 0.16 PDI for 60:40 NPs and 75:25 NPs) and had negative ζ-potential (−34.7 and −34.9, respectively). In line with the smaller size of the 60:40 formulation, a slightly lower number of HAse molecules was found per NP (244 molecules/NCs), although this was not statistically significant. In absolute terms, the enzyme content of 75:25 NPs was more similar to that of the 50:50 formulation: i.e., 335.6 molecules/NP. However, when compared with the polymer used, the loading followed an opposite pattern, i.e., 2.5 and 1.2 µg HAse/mg polymer for the 60:40 and 75:25 formulations, respectively. This was also the case for the %EE, which was highest for 60:40 NPs vs. 75:25 ones (27.9% vs. 15.0%, respectively; Table 1). 

Based on these differences, it is difficult to speculate which of the three formulations would be preferred. All NP formulations were monodispersed and had a markedly negative ζ-potential, which are favorable parameters regarding colloidal stability [52,53]. Their size range is compatible with transcellular and intracellular trafficking, including transport to lysosomes, as previously shown [31,32,33,34]. This is particularly true in the case of ICAM-1 targeting, as the associated cell adhesion molecule (CAM)-mediated pathway takes place with similar efficacy for particles up to the micrometer range [54,55,56,57]. However, smaller NPs may be preferred if other transport routes are used, such as those mediated by clathrin-coated pits or, especially, caveoli, given their greater size restrictions [57,58,59]. Regarding EE, 60:40 PLGA NPs provided an advantage and may be more favorable in terms of yield and cost associated with enzyme waste during fabrication. Yet, this may not stand true when pursuing the manufacturing of larger NP amounts, such as those needed for clinical trials and commercialization, which will use specialized procedures to sustain proper scalation and reproducibility [60,61]. On the other hand, the lower enzyme molecules held per NP in the 60:40 NP formulation may render lower activity delivered within each lysosome. However, because these NPs were also smaller, they may distribute to more lysosomes per cell and provide activity to a larger number of these compartments. If this was the case, this may compensate for their lower load and result in a similar efficacy to the slightly larger 50:50 and 75:25 NP formulations. Yet, this aspect would also depend on these formulations’ stability and release patterns, which we tested next.

### 3.2. Stability under Lysosomal Conditions of PLGA NPs Encapsulating HAse

**Figure 2:**
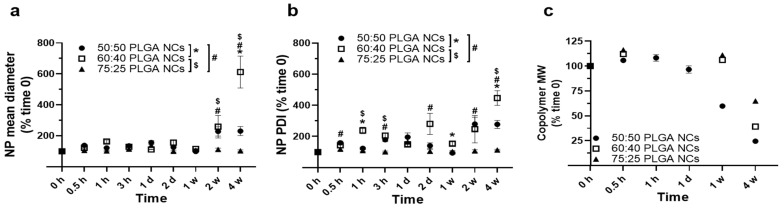
Stability of HAse-encapsulating PLGA NPs under lysosomal conditions. PLGA NPs encapsulating HAse were prepared using 50:50, 60:40, or 75:25 lactide:glycolide copolymers, and their stability was assessed under lysosomal conditions at the indicated times points by measuring (**a**) NP hydrodynamic diameter and (**b**) PDI, both using DLS, and (**c**) their polymer molecular weight (MW) using GPC. Data are mean ± SEM (*p* < 0.05 for one-way ANOVA). At each time point, * compares 60:40 to 50:50 PLGA NPs, # compares 75:25 to 50:50 PLGA NPs, and $ compares 75:25 to 60:40.

We next studied the fate of NPs under lysosomal conditions, which encompassed incubation at 37 °C and pH 4.5 in ALF, as this is the intended destination for these formulations. Regarding 50:50 PLGA NPs (Figure 2 and Supplementary Figure S1), similar to the formulation used in our previous study [32], we observed increasing and decreasing fluctuations in diameter (Supplementary Figure S1a) and PDI (Supplementary Figure S1b) for up to 1 week, which were found to be statistically significant using Student’s *t*-test but not using ANOVA. Interestingly, no fluctuations in these parameters were seen when we examined a similar formulation under physiological conditions [32]. This may be due to the acidic pH used to simulate lysosomes in this study vs. the neutral pH previously used. In fact, acidic pH has been shown to accelerate the degradation of PLGA and other biodegradable polymers used to fabricate drug NPs [62,63,64]. However, by week 2, the NP diameter increased 2.3-fold (Supplementary Figure S1a), along with a 2.8-fold increase in PDI (Supplementary Figure S1b). This indicates that, under lysosomal conditions, NPs may form small aggregates early on and mainly become unstable after 1 week, which is also consistent with the literature [65].

Interestingly, the copolymer MW was found to significantly decrease before this time (Supplementary Figure S1c), with a lower degradation of about 41% of the original MW between 1 day and 1 week and up to 76% of the original MW between weeks 1 and 4 (Supplementary Figure S1c). This agrees with previous data examining the degradation of similarly sized 50:50 PLGA NPs in vitro and in vivo [65,66]. It also indicates the existence of polymer domains more prone to fast or slow degradation, as previously reported [43], which can be affected by their location in internal pores or the particle surface [44,67].

Therefore, it appears polymer degradation starts much earlier than NP main destabilization observed after one week. This has been observed in prior studies showing a faster hydrophilic attack in internal spaces compared with the NP surface [44,67]. This causes NP internal pores to widen and particles to become hollow structures surrounded by a polymer shell, which holds the particle in place until massive polymer degradation destabilizes the whole structure [44,67].

Furthermore, we examined the stability under lysosomal conditions of the other two formulations (Figure 2). As for 75:25 PLGA NPs, they showed no relevant change in the hydrodynamic diameter or PDI over the 4 weeks studied (Figure 2a,b), indicating sustained stability. This was somewhat expected given the high lactide:glycolide ratio of this formulation, which is known to decrease PLGA hydrophilicity and, thus, its hydrolysis [43,44,45]. In agreement with this, no change in the polymer MW was observed by 1 week, and a 35% decrease was seen by week 4 (Figure 2c). This decrease seems insufficient to cause NP destabilization, just as the 41% decrease in the MW of the 50:50 PLGA copolymer observed at week 1 (Figure 2c) did not suffice to destabilize those NPs (Figure 2a,b).

Instead, we observed that NPs made from the 60:40 PLGA copolymer behaved more similarly to those fabricated from 50:50 PLGA in terms of changes in hydrodynamic size (Figure 2a). They fluctuated up and down for 1 week and then showed a significant increase, reaching 2.6-fold and 6.1-fold rises in diameter by weeks 2 and 4, respectively (Figure 2a). This was observed along with more significant fluctuations in NP PDI, which started at 1 h and peaked between weeks 2 and 4 (Figure 2b). These data contrasted with those observed for 50:50 PLGA NPs, which did not change beyond week 2 (Figure 2a,b). This would suggest that the 60:40 copolymer may take longer to degrade and, hence, this NP population may take longer to fully destabilize, although their destabilization may begin earlier. In agreement with this, the GPC analysis of polymer MW showed no change in the 60:40 polymer by week 1, while the 50:50 polymer had decreased by 41% by this time (Figure 2c). This was expected given the greater lactide:glycolide ratio of the former formulation [42,43,44]. As for destabilization beginning earlier for 60:40 PLGA NPs despite their slower polymer degradation, this may be due to their smaller diameter (Table 1) and, thus, their greater surface-to-volume ratio [44,45,48]. This may result in a greater hydrophilic attack at the NP surface and easier penetration into the inner matrix, destabilizing the shell-like intermediates that result from NP internal degradation [44,45]. 

Altogether, 75:25 PLGA NPs showed slower polymer degradation and NP destabilization, which may render more sustained enzyme release and, thus, require less frequent administration compared to the other two formulations. This is important because, typically, lysosomal ERTs are administered every two weeks in specialized medical centers, which increases the cost and burden of these treatments [11,12]. However, the more sustained stability of 75:25 PLGA NPs could also cause unwanted lysosomal storage of polymer, negatively affecting diseased cells, which already have an altered lysosomal function due to LSDs [1,2,3,4]. 

### 3.3. Role of a Targeting Antibody Coat on Stability under Lysosomal Conditions of PLGA NPs Encapsulating HAse

As stated, our previous study on PLGA NPs encapsulating lysosomal enzymes also involved NP coating with an antibody that recognizes ICAM-1 [32]. This provided specific targeting in vitro and in vivo, as well as endocytosis and lysosomal trafficking, the intended destination for these formulations [32]. Hence, we investigated whether the presence of an antibody coat on NPs would affect their stability under lysosomal conditions. 

We used NPs fabricated using the 50:50 lactide:glycolide copolymer as an example (Table 2). The anti-ICAM-1 coating increased the NP diameter from 167.8 to 224.9 nm, their PDI from 0.14 to 0.19, and their ζ-potential from −38.4 to −32.6 mV. These changes were expected, similar to those observed in our previous publication [32], and still compatible with intracellular trafficking and colloidal stability [31,32,33,34,52,53].

**Figure 3:**
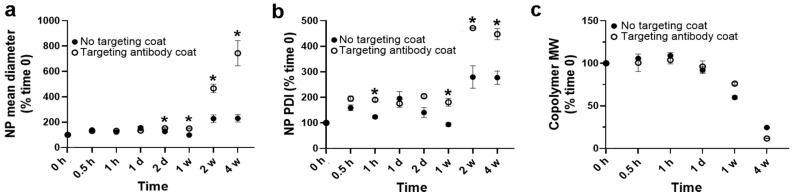
Role of a targeting antibody coat on PLGA NP stability under lysosomal conditions. PLGA NPs (50:50 lactide:glycolide copolymer) encapsulating HAse were either coated or not with anti-ICAM-1 and then their stability was assessed under lysosomal conditions at the indicated time points by measuring (**a**) NP hydrodynamic diameter and (**b**) PDI, both using DLS, and (**c**) their polymer molecular weight (MW) using GPC. Data are mean ± SEM (*p* < 0.05 using Student’s *t*-test). * Compares coated NPs vs. non-coated NPs.

Upon incubation under lysosomal conditions, antibody-coated NPs presented slightly higher changes in size by day 2 and up to 1 week (Figure 3a) compared with non-coated NPs, which was also reflected by modestly higher changes in PDI during the first week (Figure 3b). However, these changes could not be explained by a different polymer degradation pattern compared to non-coated NPs (Figure 3c). By weeks 2 and 4, we observed more acute changes between the coated and non-coated formulations (Figure 3a,b); yet, again, they could not be explained by the polymer degradation data (Figure 3c). Therefore, it is possible that under lysosomal conditions, antibody-coated NPs tend to aggregate more and faster. For instance, antibody molecules on the coat of a NP could interact with antibody molecules or the polymeric surface of neighboring NPs as a consequence of antibody degradation, denaturation, uncoating, etc. [68,69]. Despite these differences, the general pattern for NP destabilization (Figure 3a,b) and polymer degradation (Figure 3c) were very similar for both formulations, with polymer degradation visible after one week, yet most marked changes in NP diameter and PDI appearing later between weeks 2 and 4.

### 3.4. Enzyme Release under Lysosomal Conditions from PLGA NPs Encapsulating HAse

**Figure 4:**
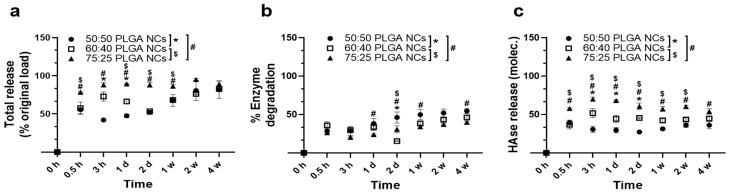
Enzyme release from HAse-encapsulating NPs under lysosomal conditions. PLGA NPs encapsulating ^125^I-HAse were prepared using 50:50, 60:40, or 75:25 lactide:glycolide copolymers and incubated under lysosomal conditions for the indicated time points. Then, the released ^125^I-HAse was separated from NPs using centrifugation and measured using a gamma counter. Data were used to calculate: (**a**) total release (^125^I counts), expressed as a percentage of the original load; (**b**) HAse degradation assessed with TCA precipitation; and (**c**) HAse molecules released in (**a**) after subtraction of degraded HAse found in (**b**). Data are mean ± SEM (*p* < 0.05 using one-way ANOVA). At each time point, * compares 60:40 to 50:50 PLGA NPs, # compares 75:25 to 50:50 PLGA NPs, and $ compares 75:25 to 60:40 PLGA NPs.

The data described above would suggest that, for 50:50 PLGA NPs, HAse may be released under lysosomal conditions in a biphasic manner: fast first, as polymer degradation starts and pores open in particles, and then when enzyme molecules remaining in the structure are released upon full NPs destabilization. We assessed this by radiotracing ^125^I-labeled HAse (Figure 4 and Supplementary Figure S2). The results showed an initial burst release of 56% of the total enzyme load within the first 30 min followed by a second release phase from weeks 1 to 4, ultimately reaching a release of 82% of the original enzyme load (Supplementary Figure S2a). This would agree with the speculation discussed above for the mechanism underlying enzyme release and the previous literature on PLGA NPs [64,70,71,72]. With regards to the other formulations, they also showed an initial burst release at 0.5 h, although they differed in several manners (Figure 4a). For instance, at this time, 50:50 and 60:40 NPs released a similar amount of cargo (55.5% and 57.5% of their original load), while the 75:25 formulation released significantly more (78.3%) and maintained this level up to week 4, where all formulations were comparable. Some differences were observed between 50:50 and 60:40 NPs at intermediate time points, e.g., 3 h and day 1, where a higher release was measured for the latter formulation (i.e., 47.5% vs. 72.8%, respectively, for day 1). Therefore, a trend was observed where enzyme release from NPs increased over time based on their higher lactide:glycolide ratio. This may be due to their increasing hydrophobicity, which may favor the release of this hydrophilic cargo [42,43]. This is similar to a previous study showing that 85:15 PLGA NPs released their rhodamine B load faster than their 65:35 counterparts when examined in vitro and in cell culture [46].

Along with the release from NPs, we also inferred the possible occurrence of enzyme degradation using TCA precipitation to separate ^125^I-HAse from free ^125^I or ^125^I attached to short peptides, as described [73]. As shown for 50:50 PLGA NPs (Figure 4b and Supplementary Figure S2b), some degradation level was found, ranging from 29% at 30 min to 55% at week 4. This early degradation level and a very slow increasing pattern suggest that alterations may rather occur during encapsulation. This may be due to the presence of solvents during NP synthesis, as previously seen [74], rather than later during the lysosomal incubation time. In fact, our previous study found a 50% decrease in HAse activity after encapsulation within this formulation [32]. Nevertheless, significant levels of the released enzyme were observed after subtracting the degraded fraction (Figure 4c and Supplementary Figure S2c). Similarly, an enzyme degraded fraction was also detected for the other formulations, which was 36% for 60:40 NPs and 26% for 75:25 NPs at 0.5 h and then slowly raised to 55% and 40%, respectively, by 4 weeks (Figure 4b). Nevertheless, significant levels of the released enzyme were observed after subtracting the degradation fraction (Figure 4c). The levels were higher for 60:40 NPs compared with the 50:50 formulation for various time points, and those of the 75:25 formulation were the highest, correlating with copolymer hydrophobicity, as discussed above.

### 3.5. Catalytic Activity of PLGA NPs Encapsulating HAse

**Figure 5:**
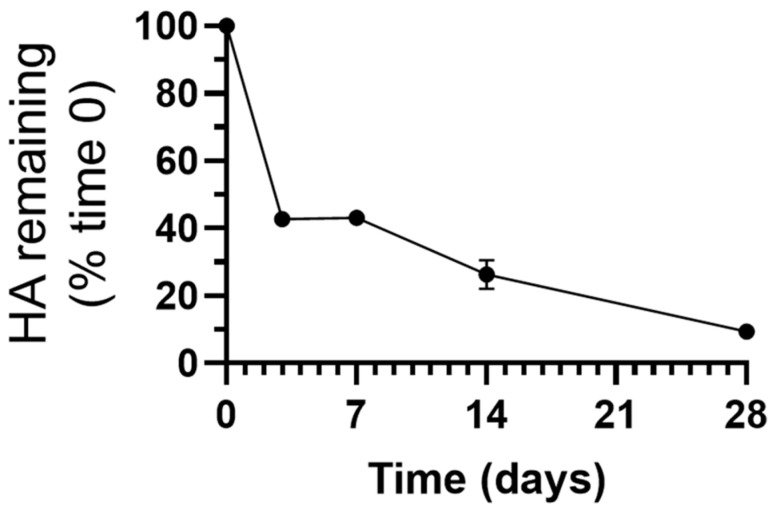
Catalytic activity of HAse-encapsulating PLGA NPs. PLGA NPs (50:50 lactic:glycolide copolymer) encapsulating HAse were incubated at 37 °C for up to 28 days in lysosomal conditions. Then, HAse catalytic activity on hyaluronic acid (HA) was measured and expressed as the percentage of HA remaining over time. Data are mean ± SEM.

Finally, we investigated whether the enzyme levels observed would hold any catalytical significance, for which we focused on the 50:50 NP formulation given its lower enzyme release level (Figure 4). Despite this fact, this formulation rendered a clear catalytic activity under lysosomal conditions, which was sustained for up to 4 weeks, the latest time tested (Figure 5 and Supplementary Figure S3). For instance, 0.11 mg HA, the enzyme substrate, was degraded by day 3 (Supplementary Figure S3), representing 57% of the total HA at the beginning of the reaction (Figure 5). This activity level was maintained by day 7, progressing until reaching 0.18 mg HA degraded by day 28 (Supplementary Figure S3), equivalent to 91% substrate (Figure 5).

These data demonstrate that HAse retains significant catalytic activity after encapsulation in PLGA NPs and is capable of sustaining activity for at least 4 weeks under lysosomal conditions, which agrees with previous results [32]. Despite the enzyme degradation level observed (Figure 4b), its original load and subsequent release were sufficient to withhold enough catalytic activity to degrade its substrate fully (Figure 5).Additionally, this activity kinetics agrees with the speculation discussed above on the mechanism underlying enzyme release and the previous literature on PLGA NPs [70,71,72] and may explain the early peak in enzyme activity observed in Supplementary Figure S3. The initial burst release and the peak in enzyme activity could also result from any enzyme adsorbed on the NP surface. However, since our previous study demonstrated that no surface enzyme was detected for this formulation [32], these events may rather be due to the fast outward diffusion of enzyme molecules loaded in the outermost part of the NP matrix, as observed for other systems [70,71,72]. The subsequent late release agrees with the continuous degradation of polymer and NP destabilization (Figure 2) and the second peak in enzyme activity (Supplementary Figure S3). Importantly, since both the 60:40 and 75:25 NP formulations released higher enzyme amounts compared to their 50:50 counterparts (Figure 4), these two formulations would be catalytically suitable since the lower enzyme levels released by 50:50 PLGA NPs were sufficient to fully degrade the HA substrate (Figure 5).

These results hold considerable relevance regarding ERT for MPS IX. This disease is caused by mutations in the *HYAL1* gene, resulting in the progressive accumulation of the extracellular matrix glycosaminoglycan, HA [48,49,50]. This leads to several skeletal alterations, primarily affecting joints and craniofacial structures, as well as the appearance of soft-tissue tumors, affecting growth, hearing, mobility, etc. [48,49,50]. Patient treatment encompasses symptomatic care, surgery, or bone marrow transplantation [48,49,50]. The results shown in this study, along with those published in reference [32], suggest that PLGA NPs could provide a new avenue for MPS IX ERT.

## 4. Conclusions

**Figure 6:**
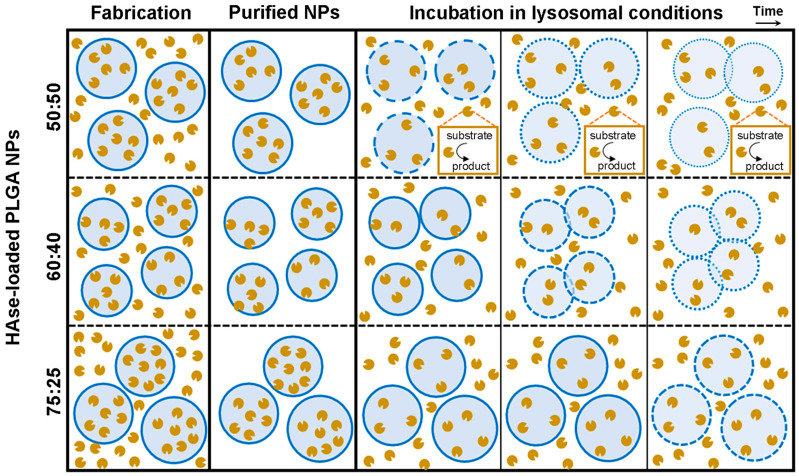
Characterization of PLGA NPs encapsulating HAse. Main physicochemical characteristics of NPs fabricated using PLGA with varying lactide:glycolide ratios, including their relative diameter, PDI, EE, and absolute enzyme load. Their relative (not drawn at scale) colloidal stability, polymer degradation kinetics, burst release, and sustained catalytic activity under lysosomal conditions are also shown over time.

The present study focused on the role of the PLGA lactide:glycolide ratio, particularly 50:50, 60:40, and 75:25, in the properties of NP encapsulating HAse, as a model for lysosomal ERT (Figure 6). All three formulations were similarly appropriate regarding size, PDI, and ζ-potential, as well as release and enzyme degradation under lysosomal conditions. They all rendered enzyme levels above those required for full substrate degradation, which was sustained for 4 weeks. However, their EE, as well as polymer degradation and NP destabilization under lysosomal conditions, varied among the three formulations. As a result, 60:40 PLGA NPs are the preferred formulation given their higher EE and fastest l destabilization under lysosomal conditions, likely due to their slightly smaller size. The 50:50 NP formulation is a good alternative due to its fastest polymer degradation and full NP destabilization while having enough loading for full substrate degradation. In contrast, 75:25 PLGA NPs are the least suitable formulation for lysosomal ERT due to their lowest EE and highest polymer and NP stability, which would render a higher cost and possible accumulation in lysosomes. Since LSDs are chronic conditions that require life-long treatment and lysosomes have an altered degradative capacity in LSDs [7,11,12], this possible NP accumulation may cause unwanted side effects. Coating NPs with a targeting antibody modestly increased their size, PDI, ζ-potential, and modestly decreased colloidal stability under lysosomal conditions. However, antibody coating did not alter the polymer degradation pattern, and NP physicochemical properties were still suitable for the intended application. Altogether, these results are relevant in guiding the design of PLGA NPs for lysosomal ERT, which was investigated here using HAse for ERT of MPS type IX but would be easily transferable to other enzymes and LSDs. Future studies will focus on examining these formulations’ therapeutic potential and side effects in patient-derived cells and animal models. In this regard, since current treatments using infusion of naked enzymes are typically administered every 2 weeks, the 4-week sustained activity provided with PLGA NPs is greatly valuable as it may extend the duration of the effects obtained after each NP administration, thus, reducing the frequency and cost of treatment.

## Figures and Tables

**Table 1:** Characterization of PLGA NPs encapsulating HAse.

PLGA Copolymer	Diameter (nm)	PDI	ζ-Potential (mV)	%EE	HAse molec./NP	µgHAse/mg Copolymer
50:50	167.8 ± 4.2	0.14 ±0.01	−38.4 ± 0.6	20.4 ± 5.7	400.9 ± 85.1	1.6 ± 0.5
60:40	121.4 ± 2.2 *	0.12 ± 0.02	−34.7 ± 1.0 *	27.9 ± 1.2	243.7 ± 35.0	2.5 ± 0.1
75:25	164.7 ± 13.1 ^$^	0.16 ± 0.02	−34.9 ± 1.8	15.0 ± 2.2 ^$^	335.6 ± 63.4	1.2 ± 0.2 ^$^

%EE = encapsulation efficiency percentage; molec. = molecule; NP = nanoparticle; PDI = polydispersity index. Data are average ± SEM. Statistical significance is *p* < 0.05. * Compares 60:40 NP or 75:25 NP to 50:50 NPs, and ^$^ compares 75:25 NPs to 60:40 NPs (one-way ANOVA).

**Table 2:** Comparison between antibody-coated and non-coated PLGA NPs encapsulating HAse.

50:50 PLGA	Diameter (nm)	PDI	ζ-Potential (mV)	HAse molec./NP	Ab molec./NP
Non-coated	167.8 ± 4.2	0.14 ±0.01	−38.4 ± 0.6	400.9 ± 85.1	ND
Ab-coated	224.9x ± 26.5 *	0.19 ± 0.02 *	−32.6 ± 2.8 *	(same as above)	187.4 ± 13.9

Ab = antibody; molec. = molecule; ND = not determined; NP = nanoparticle; PDI = polydispersity index. Data are average ± SEM. * Compares Ab-coated NPs vs. non-coated NPs (*p* < 0.05 with Student’s *t*-test).

## Data Availability

Data will be provided upon request. Unique materials can be shared upon reasonable request and at the recipient’s cost, respecting the protection of any active confidentiality and intellectual property provisions.

## Supplementary Materials

The following are available online at https://www.mdpi.com/article/10.3390/jfb14090440/s1, Figure S1. Stability of 50:50 PLGA NPs encapsulating HAse under lysosomal conditions. Figure S2. Enzyme release from 50:50 PLGA NPs under lysosomal conditions. Figure S3. Catalytic activity derived from HAse-encapsulating PLGA NPs.
