# Supplementary material for: Role of the Lactide:Glycolide Ratio in PLGA Nanoparticle Stability and Release under Lysosomal Conditions for Enzyme Replacement Therapy of Lysosomal Storage Disorders
Source: J Funct Biomater. 2023 Aug 25;14(9):440. doi: 10.3390/jfb14090440 (PMC10531859; doi:10.3390/jfb14090440)
Supplement: Supplementary file 1 [file jfb-14-00440-s001.zip › jfb-2514441-supplementary.pdf]

Role of the Lactide:Glycolide Ratio in PLGA Nanoparticle Stability and Release under Lysosomal Conditions for Enzyme Replacement Therapy of Lysosomal Storage Disorders

Maria del Moral [1,2,†], Maximilian Loeck [1,2,†], Eameema Muntimadugu [3], Guillem Vives [1,4], Vy Pham [3,5], Peter Pfeifer [1], Giuseppe Battaglia [1,6] and Silvia Muro [1,3,5,6,*]

[1] Institute for Bioengineering of Catalonia (IBEC), Barcelona Institute for Science and Technology,
08028 Barcelona, Spain; delmoral1999@gmail.com (M.d.M.); mloeck@ibecbarcelona.eu (M.L.); guillev9837@gmail.com (G.V.); ppfeifer@ibecbarcelona.eu (P.P.); gbattaglia@ibecbarcelona.eu (G.B.)

[2] Applied Materials Chemistry Master Program (M.d.M) and Biomedicine Doctorate Program, University of Barcelona, 08007 Barcelona, Spain

[3] Institute for Bioscience and Biotechnology Research (IBBR), University of Maryland, College Park, MD 20742, USA; emeemapristis@gmail.com (E.M.); vpham197@terpmail.umd.edu (V.P.)

[4] Nanoscience and Nanotechnology Degree Program, Autonomous University of Barcelona, 08193 Bellaterra, Spain

[5] Department of Chemical and Biomolecular Engineering, University of Maryland, College Park, MD 20742, USA

[6] Institution of Catalonia for Research and Advanced Studies (ICREA), 08010 Barcelona, Spain

* Correspondence: smuro@ibecbarcelona.eu

† These authors contributed equally to this work.

Keywords: Lysosomal storage disorder; enzyme replacement therapy; hyaluronidase; poly(lactide-co-glycolide) nanoparticles; copolymer ratio; nanoparticle stability; enzyme release.

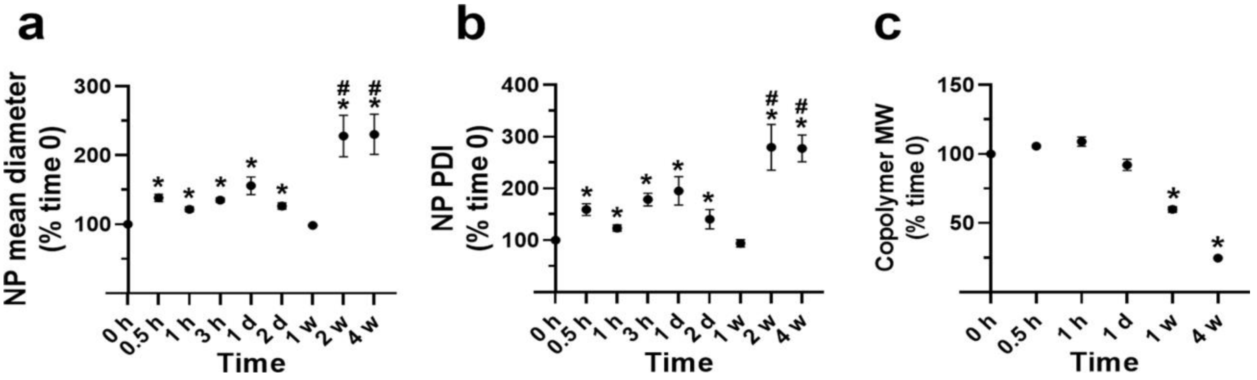

Figure S1. Stability of 50:50 PLGA NPs encapsulating HAse under lysosomal conditions. This formulation was incubated under lysosomal conditions from 0.5 hours (h), through the indicated days (d), and up to 4 weeks (w). After this incubation, stability was assessed by measuring (a) NP hydrodynamic diameter and (b) PDI, both by DLS, and (c) polymer molecular weight (MW) by GPC. Data are mean±SEM ($p < 0.05$ for statistical significance). *Compares each time point to time 0 by Student's *t* test or # one-way ANOVA.

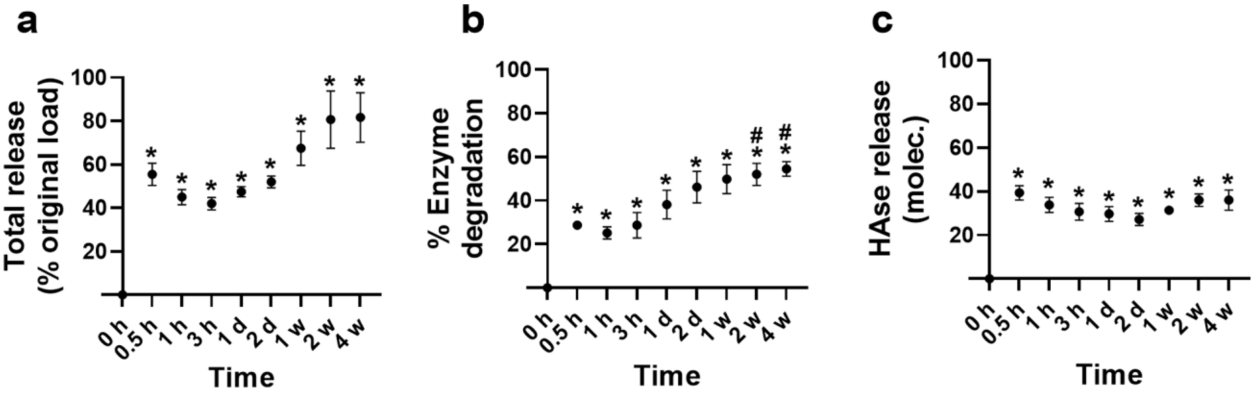

Figure S2. Enzyme release from 50:50 PLGA NPs under lysosomal conditions. The release of ^{125}I-HAse encapsulated in this formulation was assessed under lysosomal conditions from 0.5 hours (h), through the indicated days (d) and up to 4 weeks (w). After this, ^{125}I-HAse release was measured in a gamma counter after NP separation by centrifugation. Data were used to calculate: (a) total release (^{125}I counts), expressed as a percentage of the original load; (b) HAse degradation assessed by TCA precipitation; and (c) HAse molecules released in (a) after subtraction of degraded HAse found in (b). Data are mean±SEM ($p < 0.05$ for statistical significance). * Compares each time point to time 0 by Student's *t* test or # one-way ANOVA.

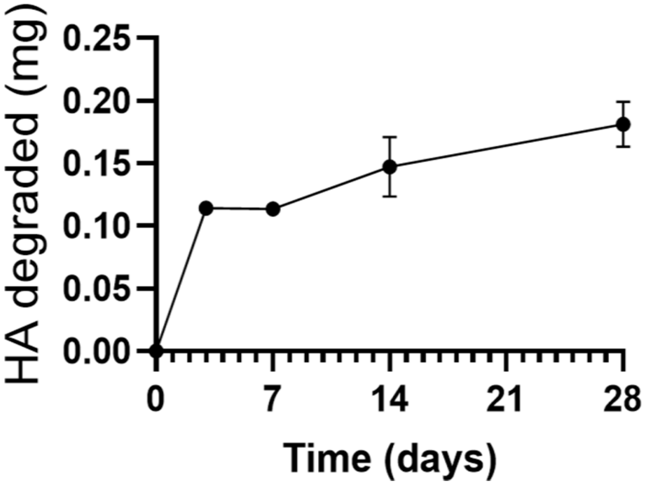

Figure S3. Catalytic activity derived from HAse-encapsulating PLGA NPs. PLGA NPs (50:50 lactic:glycolide copolymer) encapsulating HAse were incubated at 37 °C for up to 28 days in lysosomal conditions. Then, HAse catalytic activity on hyaluronic acid (HA) was measured and was expressed as the amount of HA degraded over time. Data are mean±SEM.
